# T Lymphocytes Contribute to the Control of Baseline Neural Precursor Cell Proliferation but Not the Exercise-Induced Up-Regulation of Adult Hippocampal Neurogenesis

**DOI:** 10.3389/fimmu.2018.02856

**Published:** 2018-12-11

**Authors:** Tara L. Walker, Sonja Schallenberg, Nicole Rund, Lisa Grönnert, Ruslan Rust, Karsten Kretschmer, Gerd Kempermann

**Affiliations:** ^1^Center for Regenerative Therapies Dresden (CRTD), Technische Universität Dresden, Dresden, Germany; ^2^German Center for Neurodegenerative Diseases (DZNE), Dresden, Germany

**Keywords:** T cell, regulatory T cell, adult neurogenesis, neural precursor cell, dentate gyrus, hippocampus, physical activity

## Abstract

Cross-talk between the peripheral immune system and the central nervous system is important for physiological brain health. T cells are required to maintain normal baseline levels of neural precursor proliferation in the hippocampus of adult mice. We show here that neither T cells, B cells, natural killer cells nor natural killer T cells are required for the increase in hippocampal precursor proliferation that occurs in response to physical exercise. In addition, we demonstrate that a subpopulation of T cells, regulatory T cells, is not involved in maintaining baseline levels of neural precursor proliferation. Even when applied at supraphysiological numbers, populations of both naive and stimulated lymphocytes had no effect on hippocampal precursor proliferation *in vitro*. In addition, physical activity had no effect on peripheral immune cells in terms of distribution in the bone marrow, lymph nodes or spleen, activation state or chemokine receptor (CXCR4 and CCR9) expression. Together these results suggest that lymphocytes are not involved in translating the peripheral effects of exercise to the neurogenic niche in the hippocampus and further support the idea that the exercise-induced regulation of adult neurogenesis is mechanistically distinct from its baseline control.

## Introduction

Brain plasticity relies on neuro-immunological cross-talk that communicates information about homeostatic states of the body, and deviations from these, from the periphery to the brain. A key finding in this context has been that immunity is involved not only in the response to damage and disease, but also in normal physiological processes (1). One of these is adult neurogenesis, the generation of new neurons in the brain (2–5). For example, regenerative neurogenesis in zebrafish has been shown to depend on immune activation (6). In rodents we have found that baseline adult hippocampal neurogenesis, in the absence of any pathology, is dependent on T cells (3). Immune-deficient mice exhibit reduced adult hippocampal neurogenesis, and T cell transplantation results in a return to normal levels. Remarkably, this effect is only seen with CD4^+^ and not CD8^+^ T cells. Mice lacking Th17 helper cells (which are CD4^+^/CD8^+^) also have reduced adult neurogenesis (7). Together these findings support the idea that T cells (but not B cells) play an important role in controlling adult neurogenesis, presumably without having to be physically present in the neurogenic niche.

Adult hippocampal neurogenesis is acutely regulated by physical activity, with short bouts of voluntary wheel running robustly increasing precursor cell proliferation in mice (8–10). As a side finding of our original report on the effects of T cells on adult hippocampal neurogenesis we noted that antibody-based and genetic depletion of CD4^+^ T cells did not abolish the responsiveness of the precursor cells to physical activity (3). This was an intriguing observation as a number of other manipulations (for example treatment with vascular endothelial growth factor (VEGF) and endocannabinoids) had the opposite effect, impairing the exercise-induced up-regulation, while leaving the baseline unchanged (11, 12). Together these findings suggested that there might be a fundamental distinction between the baseline *control* and activity-dependent *up-regulation* of adult hippocampal neurogenesis (13).

To address this possibility, we designed a set of experiments to explore the extent to which T cell populations are necessary for the exercise-induced increase in precursor cell proliferation in the adult mouse hippocampus. We also investigated whether T cell populations in the bone marrow and peripheral lymphoid organs respond to exercise and whether running-induced changes occur in key chemokine receptors on lymphocytes.

## Materials and Methods

### Mice

C57BL/6.Foxp3-IRES-RFP (14), T cell receptor alpha (TCRα)^−/−^ (15) and B6.Rag1^−/−^ (16) mice were purchased from The Jackson Laboratory. C57BL/6.Rag2^−/−^cγ^−/−^ (17, 18) mice were purchased from Taconic Farms and C57BL/6.CD45.1 × Foxp3^GFP^ (19) mice were originally provided by H. von Boehmer (Dana-Farber Cancer Institute, Boston, USA). Foxp3 BAC transgenic mice expressing a human diphtheria toxin receptor-GFP fusion protein (termed “*de*pletion of *reg*ulatory T cell” mice; Dereg) (20) were on the C57BL/6 background. All animals were 6–8 weeks old at the time of the experiment. For the exercise experiments, mice were housed individually with access to a running wheel for 4 days. All mice were bred and housed at the Animal Facility of the Center for Regenerative Therapies Dresden under specific pathogen-free conditions. Animal experiments were approved by the Regierungspräsidium Dresden.

### BrdU and Ki67 Immunohistochemistry and Quantification of Proliferation

To label proliferating cells, mice were given an intraperitoneal injection of 50 mg/kg bromodeoxyuridine (BrdU; Sigma), 2 h prior to perfusion. They were then perfused with NaCl (0.9% w/v) and their brains removed and stored overnight in 4% paraformaldehyde at 4°C. The following day, the brains were transferred to a 30% sucrose solution for 2–3 days. Coronal sections (40 μm) were cut using a sliding microtome (Leica SM2010) cooled with dry ice. Sections were collected and stored in cryoprotection solution (CPS) at −20°C. Every sixth section of each brain was pooled in one series for immunohistochemistry.

Briefly, brain sections stored in CPS were transferred into phosphate buffered saline (PBS) and washed. Endogenous peroxidase activity was blocked by adding 0.6% hydrogen peroxide (Merck Millipore) for 30 min, after which the sections were rinsed with 0.9% NaCl. Sections being stained with the BrdU antibody were treated with pre-warmed 1 M hydrochloric acid (Emsure) for 30 min at 37°C. After washing, the sections were blocked with a blocking solution (10% donkey serum, 0.2% Triton X-100 in PBS) for 1 h. For quantification of BrdU^+^ cells, sections were stained for BrdU (anti-rat BrdU, 1:500; AbD Serotec), followed by incubation with an anti-rat-biotin secondary antibody (1:1,000; Jackson ImmunoResearch). Ki67 staining was performed with Ki67 primary (rabbit anti-Ki67, 1:500; Novocastra) and donkey anti-rabbit-biotin secondary (1:1,000; Jackson ImmunoResearch) antibodies. Detection was performed using the Vectastain ABC-Elite reagent (Vector Laboratories) with diaminobenzidine (Sigma) and 0.04 % NiCl as the chromogen. Sections were mounted onto gelatin-coated glass slides, dried, cleared with Neoclear (Merck), and coverslipped using Neo-mount (Merck). Every sixth section (240 μm apart) was counted in the complete ventral dorsal extent of the subgranular zone (SGZ) of the dentate gyrus (DG), at 40x magnification using a standard brightfield microscope. Results were multiplied by 6 in order to obtain the total number of positive cells per brain.

### *In vivo* Depletion of Regulatory T Cells

C57BL/6.Dereg mice were intraperitoneally injected with 0.5 μg/ml diphtheria toxin (DT) in PBS or PBS only for two consecutive days. After 5 days, blood lymphocytes were isolated to determine the depletion efficiency of regulatory T cells (Tregs) in the DT-treated mice. After 7 days, mice were perfused as described above.

### Flow Cytometry and Cell Sorting

Single-cell suspensions of spleen, mesenteric lymph nodes or a pool of subcutaneous lymph nodes (*Lnn. mandibularis, Lnn. cervicales superficiales, Lnn. axillares et cubiti, Lnn. inguinales superficiales*, and *Lnn. subiliaci*) were prepared using 70 μm cell strainers (BD Biosciences). Bone marrow cells were harvested from femurs and tibias by flushing the cavities of intact bones with flow cytometry buffer (Hank's buffered salt solution, supplemented with 5% fetal calf serum and 10 mM HEPES), followed by filtration through 70 μm cell strainers. Single-cell suspensions from the spleen and bone marrow were subjected to red blood cell lysis (erythrocyte lysis buffer, Qiagen). Monoclonal antibodies to CD4 (RM4-5, GK1.5), CD8 (53–6.7), CD19 (1D3), CD25 (PC61, 7D4), CD69 (H1.2F3), CXCR4 (2B11), CCR7 (4B12), CCR9 (CW-1.2), CD62L (MEL-14), and CD44 (IM 7) and PE- and PE-Cy7-conjugated streptavidin were obtained from ThermoFisher (eBioscience) or BD Biosciences. Before flow cytometry, for some experiments, CD4^+^ or CD25^+^ cells were enriched from single-cell suspensions using biotinylated antibodies directed against CD4 or CD25, respectively, streptavidin-conjugated microbeads, and the AutoMACS magnetic cell separation system (Miltenyi Biotec). Samples were analyzed on an LSRII or LSR Fortessa system (BD Biosciences). Data were analyzed using FlowJo software (Tree Star).

### T Cell Culture for Neurosphere Assay

T cells were cultured in 96-well round-bottom plate (Greiner) in the presence of 200 μl RPMI 1640 supplemented with 10% fetal calf serum (v/v), 1 mM sodium pyruvate, 1 mM HEPES, 2 mM Glutamax, 100 U/ml Penicillin-Streptomycin, 0.1 mg/ml Gentamicin, 0.1 mM non-essential amino acids, and 0.55 mM β-mercaptoethanol (all Invitrogen). For *in vitro* activation, CD4^+^ T cells, naïve T cells (CD4^+^CD62L^high^CD25^−^) or Tregs (CD4^+^Foxp3^GFP+^) were cultured in the presence of 10 μg/ml plate-bound anti-CD3e (145-2C11), 2 μg/ml soluble anti-CD28 (37.51), and 100 U/ml human interleukin-2 (Teceleukin, Hoffmann-La Roche). The cells were cultured at a density of 7.5 × 10^4^ per well, and harvested after 3 days.

### Neurosphere Culture

Mice (8 weeks old) were killed, their brains immediately removed, and the DG microdissected (21, 22). The tissue was enzymatically digested using the Neural Tissue Dissociation Kit (Miltenyi) according to the manufacturer's instructions. Following a final wash in Hank's balanced salt solution (GE Healthcare) the pellet was resuspended in 1 ml of neurosphere growth medium and filtered through a 40 μm cell sieve (Falcon; BD Biosciences). Hippocampal cells were seeded into the wells of a 24-well plate and ~400,000 T cells were placed in a transwell insert (Merck) over these cells. After 2 days of co-culture the T cells were removed and the hippocampal cells cultured for an additional 10 days to allow neurosphere formation, after which the neurospheres were counted and measured.

### Statistical Analysis

Comparisons were made using either a one-way ANOVA with a Dunnett's *post-hoc* test, a two-tailed Mann Whitney or a Student's *t*-test using Prism 6 software (GraphPad Software), as indicated in the individual figure legends. All data presented in the text is provided as mean ± standard error. All raw data for the results presented in this manuscript can be found in the Supplementary Data section.

## Results

### T Cell-Dependent Control of Baseline Proliferation Does Not Involve Regulatory T Cells

**Figure 1:**
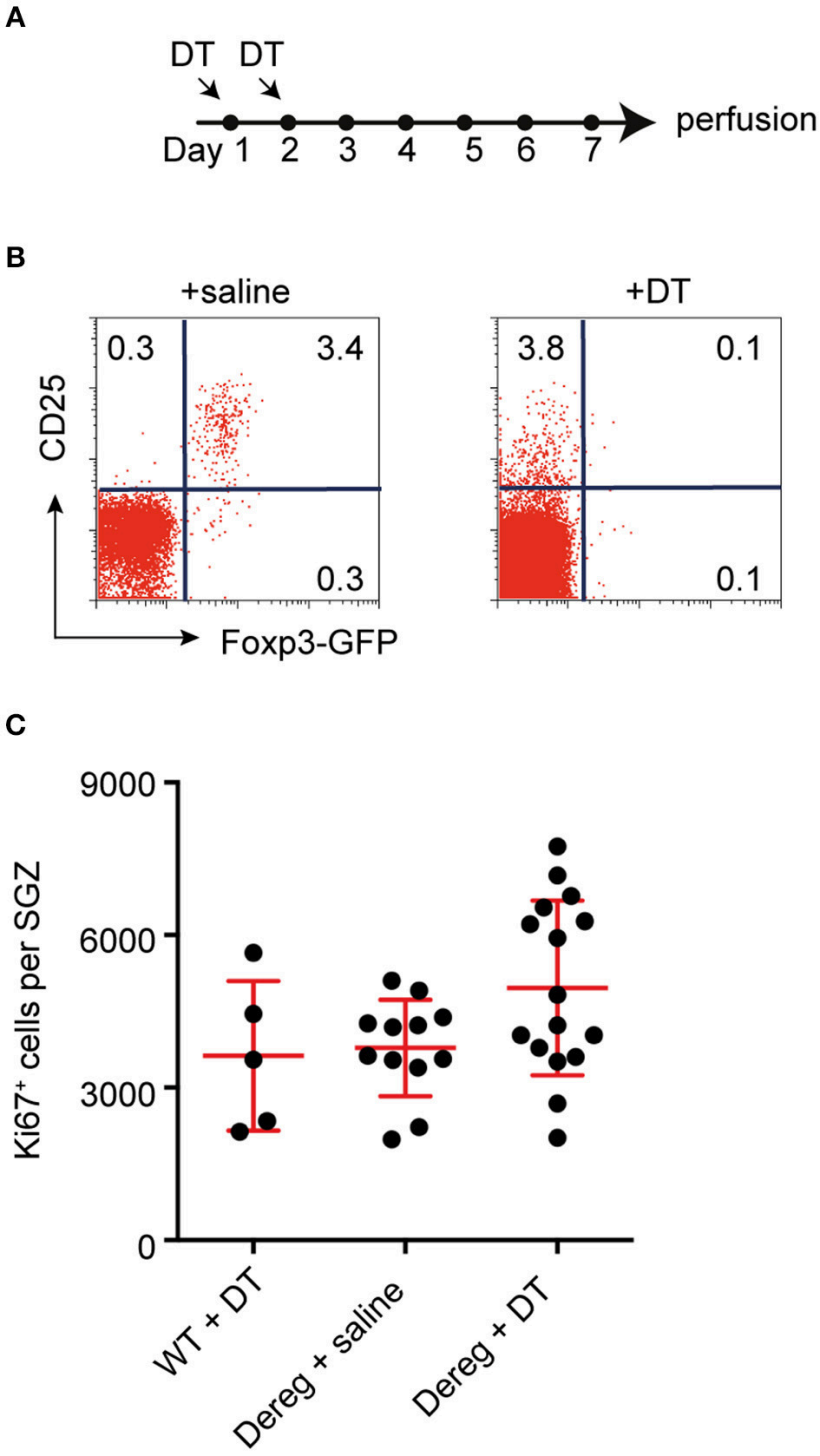
Tregs are not required to maintain baseline levels of hippocampal neurogenesis. **(A)** Experimental design. **(B)** Representative dot plots of the frequencies of CD25^+^ Foxp3-GFP^+^ Treg cells among gated CD4^+^ T cells in the blood of saline- and DT- treated B6.Dereg mice. **(C)** Depletion of Tregs had no effect on the number of proliferating (Ki67^+^) precursor cells observed in the hippocampal SGZ. Data were analyzed using a one-way ANOVA with a Dunnett's *post-hoc* test. Symbols and horizontal lines indicate individual mice and mean values ± SEM, respectively.

It has been previously reported that T cell-deficient mice have deficits in hippocampal neurogenesis (3, 7), and that depletion of one particular subset of T cells, Th17 helper cells, is sufficient to decrease hippocampal precursor proliferation under baseline conditions (7). To corroborate the specificity of the latter finding we investigated regulatory T cells (Tregs), another important subpopulation of T cells that, among other functions, has been shown to be important for functional brain recovery following stroke (23). In line with this, activated Tregs have also been reported to increase proliferation in the neurogenic subventricular zone (SVZ) of both normal and ischemic mice (24). We therefore investigated whether Tregs are involved in maintaining baseline neurogenesis in the other neurogenic niche, the hippocampus. To deplete Tregs, C57BL/6.Dereg mice, or their wild-type (WT) littermates, were injected with DT or saline for two consecutive days (Figure 1A). Five days after the second injection, we confirmed that the transient depletion of Tregs was successful in only the Dereg + DT treatment group, and demonstrated that the Treg compartment had started to recover (Figure 1B). The number of proliferating cells in the DG was then quantified by Ki67 staining. We found no significant difference in the number of proliferating precursor cells between the Treg-depleted mice (Dereg + DT: 4961 ± 428.7 Ki67^+^ cells) and their non-depleted littermates (WT + DT: 3626 ± 658.3 and Dereg + saline: 3783 ± 274.1 Ki67^+^ cells; Figure 1C). In addition, although the variance in the number of Ki67^+^ cells observed in the Dereg + DT group appeared to be larger than that in the Dereg + saline group, this result was not statistically significant (Bartlett's test for equal variances; *p* = 0.15). Together with our previous data these results suggest that Th17 helper cells but not Tregs are involved in the baseline control of precursor cell proliferation during adult hippocampal neurogenesis. Given that Tregs play critical roles in suppressing immunity, this in turn further implies that a physiological, yet nominally “pro-inflammatory” response underlies the control of baseline neural precursor proliferation.

### Lymphocytes Are not Required for the Exercise-Induced Increase in Hippocampal Precursor Proliferation

**Figure 2:**
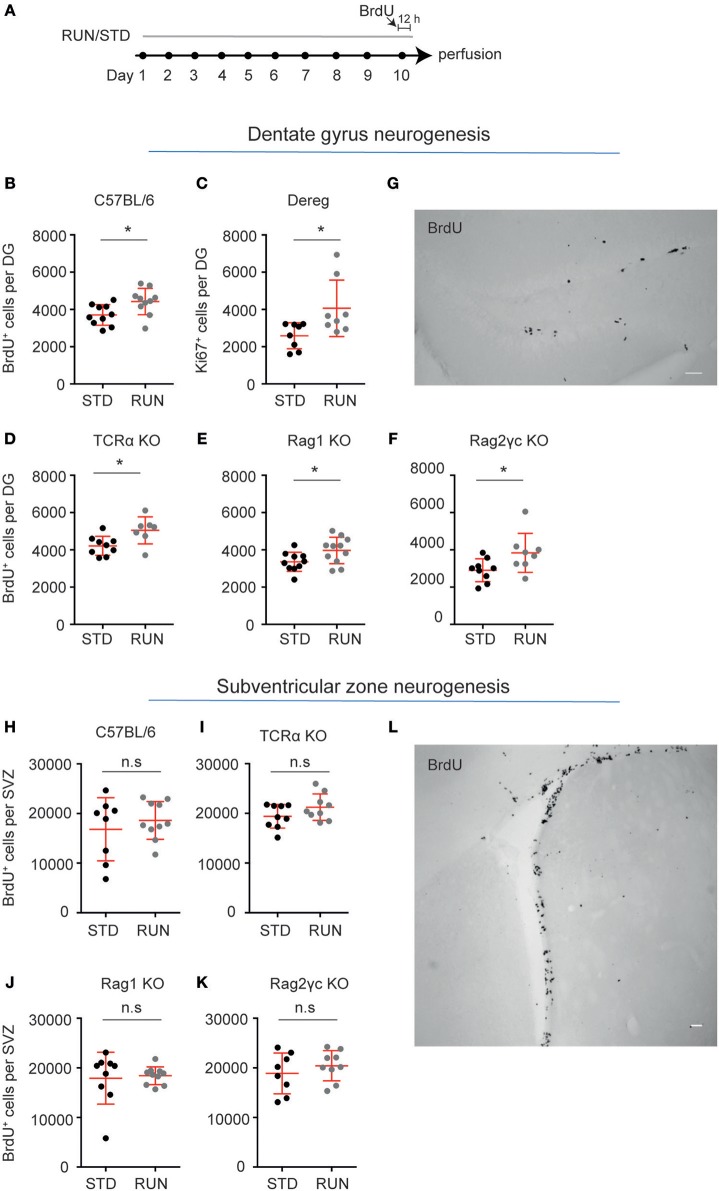
Lymphocytes are not required for the exercise-induced increase in hippocampal precursor proliferation**. (A)** Experimental design. Following exercise, a significant increase in the number of proliferating precursor cells was observed within the DG of C57BL/6 **(B)**, Dereg **(C)**, TCRα^−/−^ **(D)**, Rag1^−/−^ **(E)**, and Rag2^−/−^γc^−/−^ **(F)** mice. **(G)** Representative image of BrdU staining in the DG of Rag2^−/−^γc^−/−^ mice. No change in baseline proliferation, or a response to physical exercise was observed in the other neurogenic niche, the SVZ, in C57BL/6 **(H)**, TCRα^−/−^ **(I)**, Rag1^−/−^ **(J)**, and Rag2^−/−^cγ^−/−^ **(K)** mice. **(L)** Representative image of BrdU staining in the DG of Rag2^−/−^cγ^−/−^ mice. Symbols and horizontal lines indicate individual mice and mean values ± SEM, respectively. The level of significance was determined using a two-tailed Student's *t*-test. ^*^p < 0.05, n.s = not significant. Scale bars in **(F,K)** are 50 μm.

We have previously reported that CD4^+^ T cell-deficient mice (depleted using either anti-CD4 antibody or CD4^−/−^ transgenics) still respond to the pro-neurogenic effect of physical activity (3), despite their lowered baseline neural precursor proliferation. However, this effect is absent in mice with a combined deficiency of T, B, and natural killer (NK) cells (3). In order to determine which population of immune cells is required for the pro-proliferative effect of exercise on hippocampal precursor cells, we exposed a number of transgenic mouse strains, in which increasing numbers of lymphocyte populations are depleted, to 10 days of physical activity (Figure 2A).

We first showed that similar to C57BL/6 mice, which exhibited significantly increased hippocampal precursor proliferation following 10 days of exercise (Figure 2B; standard-housed (STD): 3703 ± 174.9 vs. RUN: 4424 ± 224 BrdU^+^ cells, *n* = 10 mice per group, *p* = 0.02), mice with depleted Tregs displayed an increase in neurogenesis in response to running (Figure 2C; STD: 2586 ± 246.4, *n* = 8 mice vs. RUN: 4062 ± 536.0 Ki67^+^ cells, *n* = 8 mice, *p* = 0.02). Using TCRα^−/−^ mice we next confirmed our previous findings (3). TCRα^−/−^ mice displayed enhanced proliferation in response to physical activity (Figure 2C; STD: 4213 ± 170.9, *n* = 9 mice vs. RUN: 5042 ± 275 BrdU^+^ cells, *n* = 7 mice, *p* = 0.02). To investigate whether B cells, which are not involved in the baseline control of neurogenesis (3), contribute to regulating the exercise-induced response, we then used Rag1^−/−^ mice which are both T and B cell deficient. Similar to WT and T cell-deficient mice, the Rag1^−/−^ mice exhibited a significant increase in hippocampal precursor proliferation following exercise (Figure 2D; STD: 3354 ± 162.5, *n* = 10 mice vs. RUN: 3969 ± 214.4 BrdU^+^ cells, *n* = 11 mice, *p* = 0.04). Finally, to investigate whether depletion of all lymphocyte populations abolished the physical activity-induced increase in hippocampal neurogenesis, as suggested previously, we used Rag2^−/−^γc^−/−^ mice (which are deficient in T cells, B cells, NK cells, and NK T cells). The Rag2^−/−^γc^−/−^ mice appeared to have a decrease in baseline hippocampal precursor proliferation compared to C57BL/6 mice, although this cannot be definitively stated as these experiments were performed on age-matched C57BL/6 mice rather than wild-type littermates. However, proliferation in the Rag2^−/−^γc^−/−^ mice still increased in response to physical activity (Figures 2E,F; STD: 2903 ± 203.9 *n* = 9 mice vs. RUN: 3839 ± 370.5 BrdU^+^ cells, *n* = 8 mice, *p* = 0.04). Interestingly, the C57BL/6 wild-type mice and all lymphocyte-depleted transgenic lines showed a similar percentage increase in proliferation following physical activity (Table 1).

**Table 1:** All lymphocyte-depleted transgenic lines showed a similar percentage increase in proliferation following physical activity.

**Population depleted**	**Strain name**	**Mean ± SEM standard**	**Mean ± SEM run**	**Difference between means**	**% increase (RUN vs. STD)**	**95% confidence interval**
none	C57BL/6	3,703 ± 174.9	4,424 ± 224.0	721.2	19.5	124.2–1318
Treg	Dereg KO	2,586 ± 246.4	4,062 ± 536.0	1476.0	57.1	210.8–2741
T cells	TCRα KO	4,213 ± 170.9	5,042 ± 275.0	828.4	19.7	164.5–1492
B cells and T cells	Rag1 KO	3,354 ± 162.5	3,969 ± 214.4	614.7	18.3	42.71–1187
B cells, T cells, NK cells, and NK T cells	Rag2γc KO	2,903 ± 203.0	3,839 ± 370.5	935.8	32.2	61.99–1810

Physical activity has been shown to increase precursor cell proliferation in the hippocampal DG (8–10, 25), but to exert no effect on precursor cells in the other major adult neurogenic niche, the SVZ (26). We therefore investigated whether lymphocyte deficiency affected the proliferation of precursor cells in the SVZ and whether such deficiency altered the ability of the SVZ precursor cells to respond to exercise. Similar to the result obtained in WT mice (Figure 2G; STD: 16834 ± 2250, *n* = 8 mice vs. RUN: 18613 ± 1205 BrdU^+^ cells, *n* = 10 mice, *p* = 0.47), deficiency in T cells (Figure 2H; STD: 194134 ± 791.2 BrdU^+^ cells, *n* = 9 mice vs. RUN: 21243 ± 886.6 BrdU^+^ cells, *n* = 9 mice, *p* = 0.47), T and B cells (Figure 2I; STD: 17913 ± 1743 BrdU^+^ cells, *n* = 9 mice vs. RUN: 18402 ± 568.3 BrdU^+^ cells, *n* = 10 mice, *p* = 0.14), and all lymphocytes (Figures 2J,K; STD: 18879 ± 1453 BrdU^+^ cells, *n* = 8 mice vs. RUN: 20419 ± 1014 BrdU^+^ cells, *n* = 9 mice, *p* = 0.39) had no effect on either baseline proliferation levels or the response to physical exercise.

### Co-culture With T Cells Does Not Affect Hippocampal Neural Precursor Proliferation

**Figure 3:**
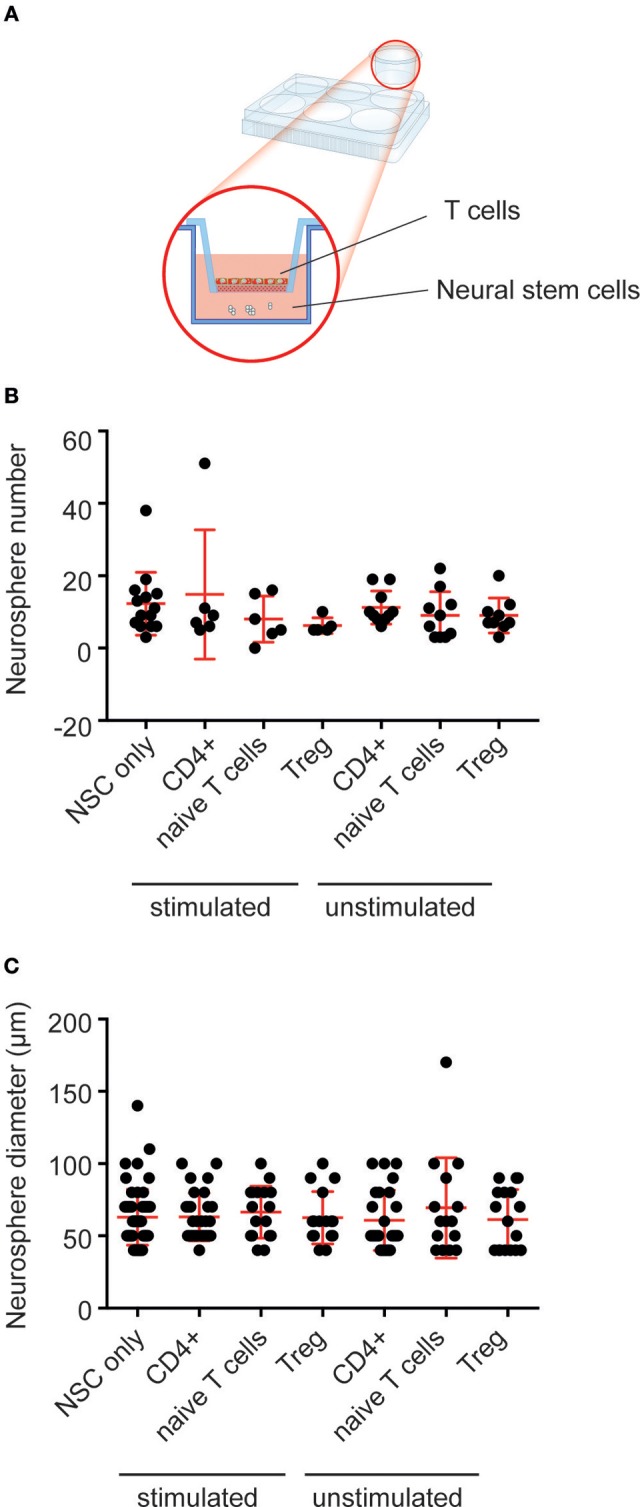
Co-culture with T cells does not affect hippocampal neural precursor proliferation. **(A)** Depiction of the experimental set-up with T cells placed in trans-well inserts over primary hippocampal cells, which are cultured in the neurosphere assay. **(B)** No difference in neurosphere number was observed following co-culture of primary hippocampal cells with populations of T cells. **(C)** Co-culture with T cells had no effect on the size of the resulting neurospheres. Symbols and horizontal lines indicate individual neural stem cell preparations and mean values ± SEM, respectively. Data were analyzed using a one-way ANOVA with a Dunnett's *post-hoc* test.

We have shown that peripheral immune cells (mast cells), which are present at relatively small numbers within the brain, can affect neural precursor proliferation *ex vivo*, when co-cultured at supraphysiological numbers (27). Similarly, very few T cells are present in the brain parenchyma (3). To determine whether exposing neural precursor cells to large numbers of purified T cell populations (~4 × 10^5^ cells) could affect neural precursor proliferation *in vitro* we used a transwell co-culture system (Figure 3A). Using flow cytometry, we sorted three populations of T cells (CD4^+^ T cells, CD4^+^CD62L^high^CD25^−^Foxp3GFP^−^ naive T cells and CD4^+^Foxp3^GFP+^ Tregs). A subset of each population was then activated, and the activation status confirmed using flow cytometry (Figure S1). Following co-culture for 2 days, the T cells were removed and the neural precursor cells were cultured for a further 10 days to allow neurosphere formation. Our analysis revealed no effect on the number of neurospheres that was generated across conditions (Figure 3B; neural precursor cells only: 12.3 ± 2.3 vs. stimulated CD4^+^ T cells: 14.8 ± 7.3 vs. stimulated naive T cells: 8 ± 2.6 vs. stimulated Tregs: 6.2 ± 0.9 vs. unstimulated CD4^+^ T cells: 11.2 ± 1.5 vs. unstimulated naive T cells: 9 ± 2.1 vs. unstimulated Tregs: 9 ± 1.6 neurospheres; *n* = 3 experiments, with between 2 and 5 wells per T cell population, *p* = 0.55). Neurosphere size gives an indication of the proliferative capacity of the precursor cell from which each neurosphere was generated. We also observed no change in neurosphere diameter following co-culture with the various T cell populations (Figure 3C; neural precursor cells only: 62.9 ± 2.5 vs. stimulated CD4^+^ T cells: 63.1 ± 3.1 vs. stimulated naive T cells: 66.5 ± 4.4 vs. stimulated Tregs: 62.5 ± 4.5 vs. unstimulated CD4^+^ T cells: 60.8 ± 4.2 vs. unstimulated naive T cells: 69.3 ± 9.0 vs. unstimulated Tregs: 61.2 ± 5.1 μm diameter; *n* = 3 experiments, with between 15 and 59 neurospheres sized per population, *p* = 0.89). This suggests that the various lymphocyte populations do not exert direct effects on hippocampal precursor cells.

### Running Does Not Have an Impact on Lymphocyte Distribution in Bone Marrow or Peripheral Lymphoid Organs

**Figure 4:**
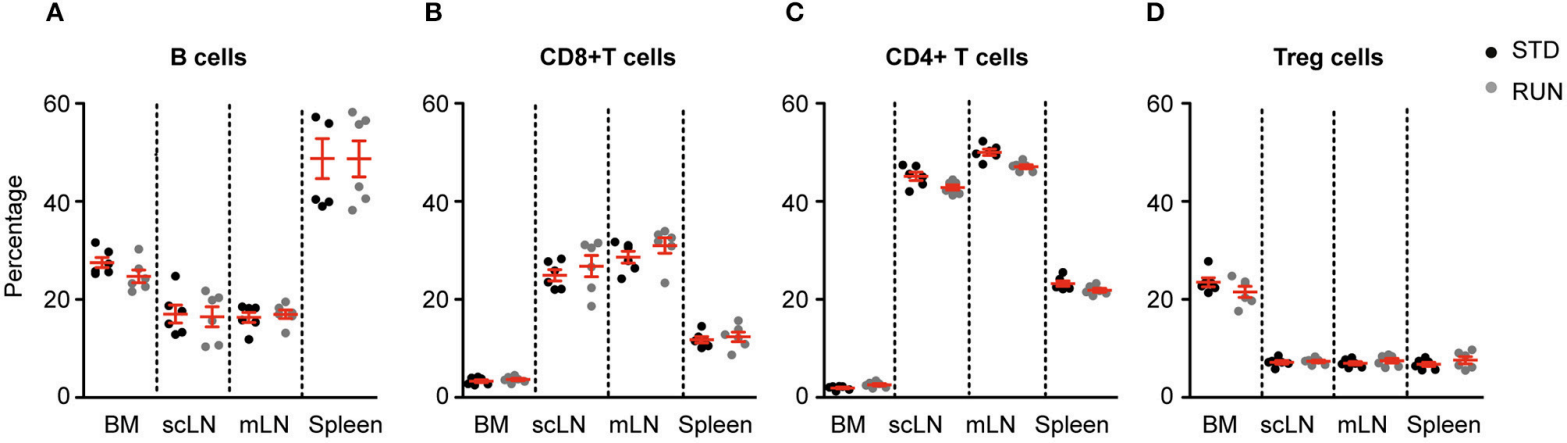
Running does not influence lymphocyte distribution in the bone marrow or peripheral lymphoid organs. Physical exercise did not change the distribution of B cells **(A)**, CD8^+^ T cells **(B)**, CD4^+^ T cells **(C)** or Tregs **(D)** in the bone marrow, lymph nodes or spleen. Mesenteric lymph nodes (mLN), subcutaneous lymph nodes (scLN). Symbols and horizontal lines indicate individual mice and mean values ± SD, respectively. Data were analyzed using a two-tailed Mann Whitney test.

**Figure 5:**
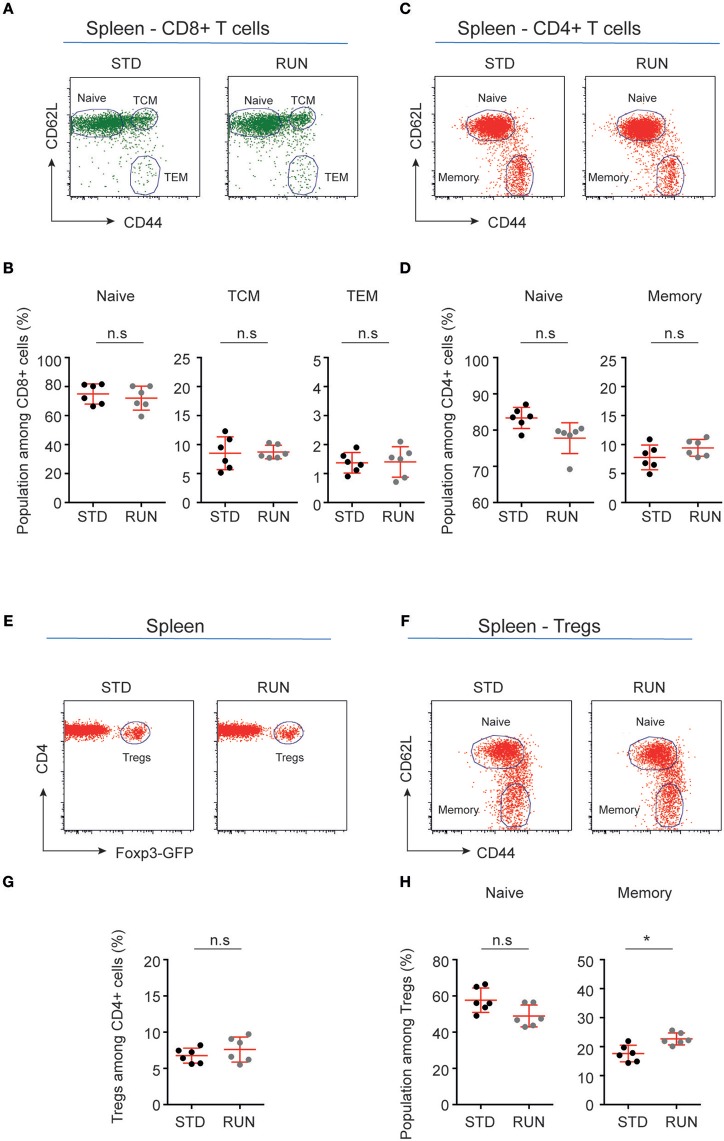
Maturation stages of CD8^+^ and CD4^+^ T cells and Tregs in the spleen of standard-housed and exercising mice**. (A,C)** Representative dot plots of CD62L and CD44 expression on CD8^+^ **(A)** and CD4^+^ **(C)** T cells in the spleen of mice that were housed under STD or RUN conditions. **(B)** Composite frequencies of naive, central memory (TCM), and effector memory (TEM) T cells among CD8^+^ T cells in the spleen. **(D)** Composite frequencies of naïve and memory T cells among CD4^+^ T cells in the spleen. **(E)** Representative dot plots depicting the expression of Foxp3-GFP among gated CD4^+^ T cells in the spleen of mice that were housed under STD or RUN conditions. **(G)** Composite frequencies of Foxp3^+^ Tregs among CD4^+^ T cells in the spleen. **(F)** Representative dot plots depicting the expression of the maturation markers CD62L and CD44 on Foxp3^+^ Tregs. **(H)** Composite percentages of naive and memory Treg cells in the spleen of STD and RUN mice after 4 days of running. Numbers in dot plots in **(B,D,G,H)** indicate the percentages of cells within the respective gate. Symbols and horizontal lines indicate individual mice and mean values ± SD, respectively. The level of significance was determined by a two-tailed Mann Whitney test. Data were compiled from two independent experiments. ^*^p < 0.05.

**Figure 6:**
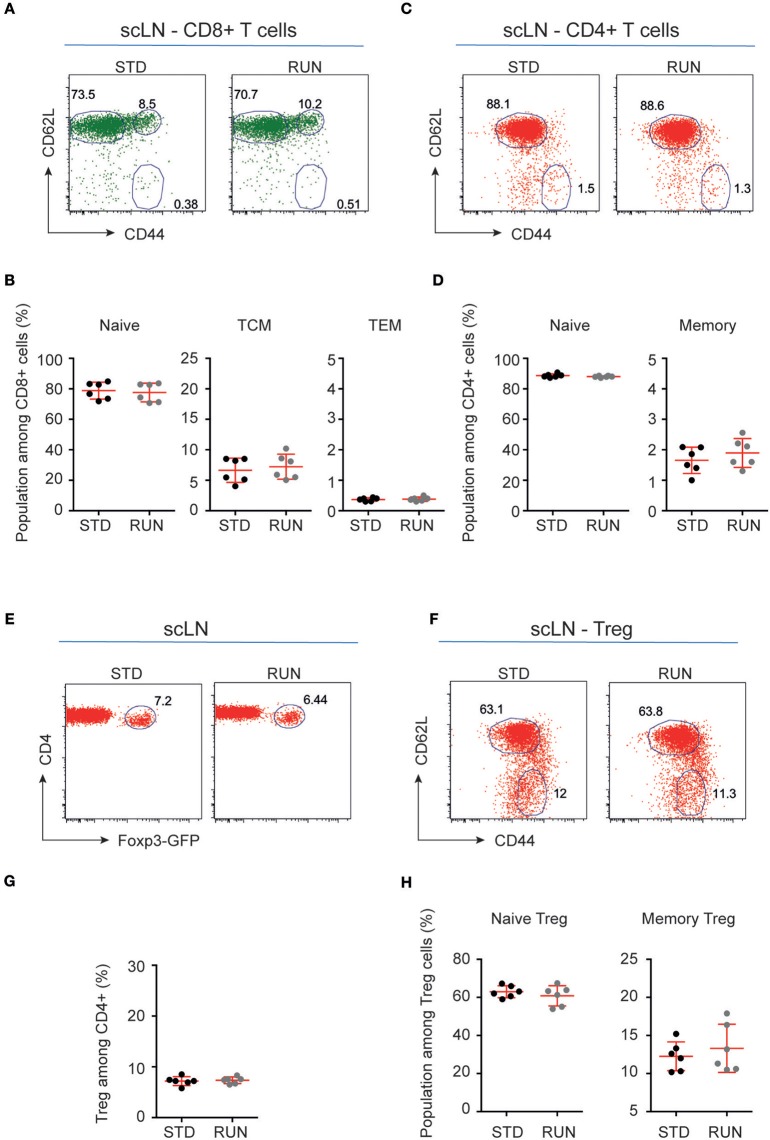
Maturation stages of CD8^+^ and CD4^+^ T cells and Tregs in the subcutaneous lymph nodes of STD and RUN mice**. (A,C)** Representative dot plots of CD62L and CD44 expression on CD8^+^ **(A)** and CD4^+^ **(C)** T cells in the subcutaneous lymph nodes (scLN) of mice that were housed under STD or RUN conditions. **(B)** Composite frequencies of naive, central memory (TCM) and effector memory (TEM) T cells among CD8^+^ T cells in the scLN. **(D)** Composite frequencies of naive and memory T cells among CD4^+^ T cells in the scLN. **(E)** Representative dot plots depicting the expression of Foxp3-GFP among gated CD4^+^ T cells in the scLN of mice that were housed under STD or RUN conditions. **(G)** Composite frequencies of Foxp3^+^ Tregs among CD4^+^ T cells in the scLN. **(F)** Representative dot plots depicting the expression of the maturation markers CD62L and CD44 on Foxp3^+^ Tregs. **(H)** Composite percentages of naive and memory Tregs in the scLN of STD and RUN mice after 4 days of running. Numbers in dot plots in **(B,D,G,H)** indicate the percentages of cells within the respective gate. Symbols and horizontal lines indicate individual mice and mean values ± SD, respectively. The level of significance was determined by a two-tailed Mann Whitney test. Data were compiled from two independent experiments.

We next investigated whether the numbers and distribution of lymphocytes would acutely respond to the running stimulus. In human subjects the massive stimulus of running a marathon, for example, is associated with a leukocytosis (28), suggesting that such an effect might be possible. To determine the effect of physical activity on the frequency of T and B cells, mice were housed under RUN or STD conditions for 4 days. Single-cell suspensions from the bone marrow, lymph nodes (subcutaneous, mesenteric), and spleen of these mice were prepared and the frequencies of CD4^+^ and CD8^+^ T cells, CD4^+^Foxp3^+^ Tregs and B cells were determined. We found that neither the frequencies nor the numbers of CD4^+^ T cells, CD8^+^ T cells, Tregs or B cells in the bone marrow, lymph nodes or spleen were influenced by running (Figure 4). We next examined whether exercise induced any changes in the phenotype of T cells. Naive T cells are characterized by the expression of high levels of CD62L and low levels of CD44, and usually home to lymph nodes. As they do not have any specific antigen contact, they need a stronger signaling to be activated. In contrast, memory T cells are antigen-experienced and partially down-regulate CD62L and express high levels of CD44. They can easily be reactivated by a repeated antigen contact and start their reaction much faster than naive T cells. Within the memory T cell subset, a further distinction can be made between central and effector memory T cells. Central memory T cells (TCM cells) express high levels of CD62L and are able to home to secondary lymphoid organs and stimulate dendritic cells. In contrast, effector memory T cells (TEM) express low levels of CD62L, home to the periphery and can very rapidly express effector cytokines following their reactivation. When we determined these phenotypes within the T cell populations after running, we observed no significant changes in the proportions of naive or memory T cells in the CD8^+^ or CD4^+^ T cell compartments in the spleen following physical activity (Figures 5A–D). However, we found a marginal but non-significant decrease in naive Tregs (Figures 5E,F), and at the same time a small but significant increase in memory Tregs in the spleen in response to exercise (Figures 5G,H). We observed no differences in the maturation stages of T cells in the subcutaneous lymph nodes after running (Figure 6).

### Increased Proportions of Naive and Decreased Percentage of Effector Memory CD8^+^ T Cells Are Present in the Bone Marrow After Physical Activity

**Figure 7:**
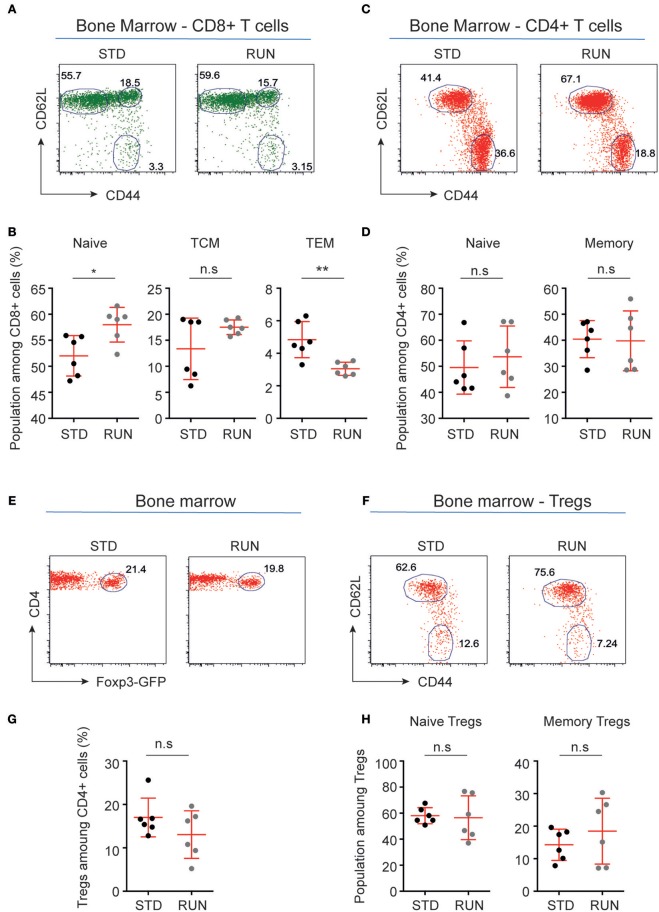
Maturation stages of CD8+ and CD4+ T cells and Tregs in the bone marrow of STD and RUN mice. **(A,C)** Representative dot plots of CD62L and CD44 expression on CD8^+^ **(A)** and CD4^+^ **(C)** T cells in the bone marrow of mice that were housed under STD or RUN conditions. **(B)** Composite frequencies of naive, central memory (TCM), and effector memory (TEM) T cells among CD8^+^ T cells in the bone marrow. **(D)** Composite frequencies of naive and memory T cells among CD4^+^ T cells in the bone marrow. **(E)** Representative dot plots depicting the expression of Foxp3-GFP among gated CD4^+^ T cells in the bone marrow of mice that were housed under STD or RUN conditions. **(G)** Composite frequencies of Foxp3^+^ Tregs among CD4^+^ T cells in the bone marrow. **(F)** Representative dot plots depicting the expression of the maturation markers CD62L and CD44 on Foxp3^+^ Tregs. **(H)** Composite percentages of naive and memory Tregs in the bone marrow of STD and RUN mice after 4 days of running. Numbers in dot plots in **(B,D,G,H)** indicate the percentages of cells within the respective gate. Symbols and horizontal lines indicate individual mice and mean values ± SD, respectively. The level of significance was determined by a two-tailed Mann Whitney test. Data were compiled from two independent experiments. ^*^p < 0.05, ^**^p < 0.01.

We next investigated whether physical activity altered the activation state of peripheral T cells. To do this, we again housed mice in either STD or RUN conditions, after which we isolated the bone marrow and performed flow cytometric analyses to determine the activation and maturation state of the T cell populations. No significant differences in naive or memory cells were detected within the CD4^+^ or CD8^+^ T cell compartment (Figures 7A–H). In contrast, we found an increase in naive T cells (STD: 52.02 ± 1.59 %, *n* = 6 vs. RUN: 57.98 ± 1.36 %, *n* = 6, *p* = 0.017) and a decrease in effector memory T cells (STD: 4.84 ± 0.45 %, *n* = 6 vs. RUN: 3.05 ± 0.16 %, *n* = 6, *p* = 0.0087) among the CD8^+^ T cells in the bone marrow (Figure 7B).

### Expression of the Cytokine Receptors CCR9 and CXCR4 Does Not Change in Response to Physical Activity

**Figure 8:**
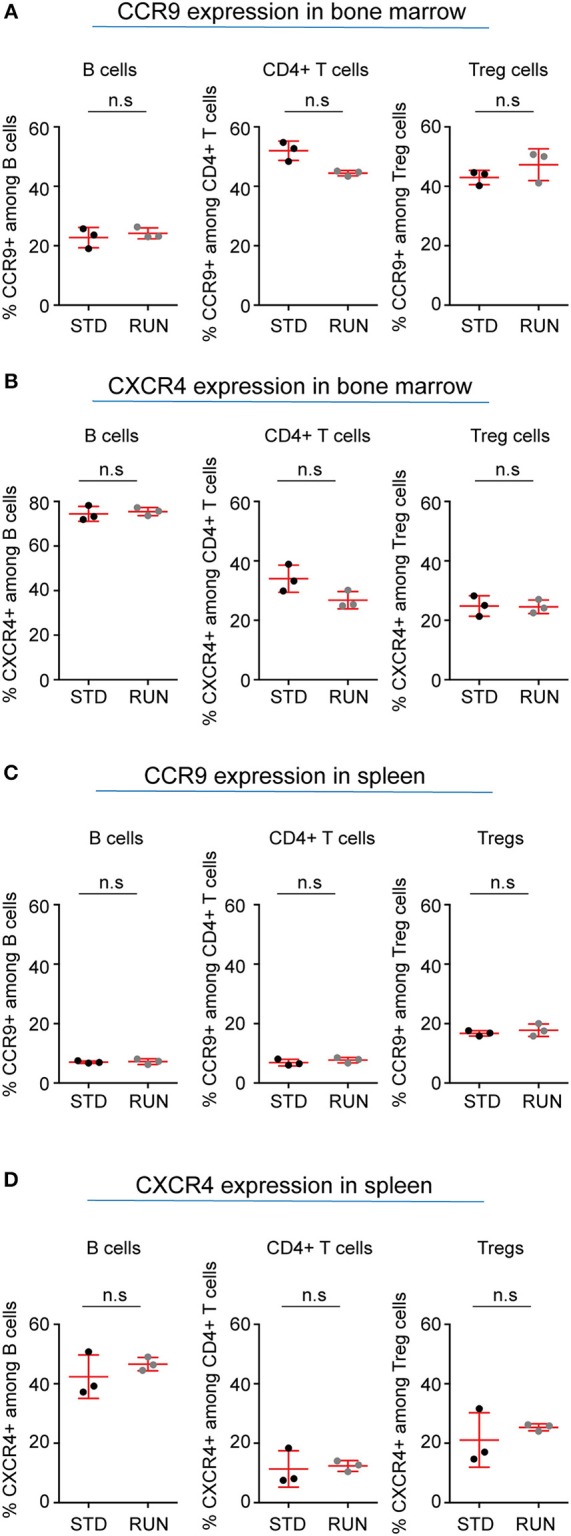
Expression of selected chemokine receptors on B cells, CD4^+^ T cells and Tregs in the bone marrow and spleen of STD and RUN mice. Composite percentages of **(A)** CCR9 and **(B)** CXCR4 expression on the indicated cell population in the bone marrow. Composite percentages of **(C)** CCR9 and **(D)** CXCR4 expression on the indicated cell populations in the spleen. Symbols and horizontal lines indicate individual mice and mean values, respectively. Data are representative of two independent experiments. Data were analyzed using a two-tailed Mann Whitney test.

Our recent finding that adult hippocampal neural stem cells express a number of cytokines and their receptors (29) has strengthened the idea of cross-talk between the immune and nervous systems. In addition, an important role of β2-adrenergic receptor signaling in controlling lymphocyte trafficking has been described (30). This effect is dependent on signaling of the chemokine receptors CCR7 and CXCR4 in lymphocytes, and further demonstrates the importance of chemokine signaling in the interaction of the immune and nervous systems (31). CCR9 is a chemokine receptor that is highly expressed on thymocytes and has also been reported to be involved in the recruitment of T cells to non-lymphoid sites such as the intestine (32). CXCR4 is expressed on a wide range of immune cells as well as on neuronal cells and mediates blood cell migration. For instance, recent studies [reviewed in (33)] showed that chemokine signaling is involved in the cross-talk between sympathetic nerves and the stem cell niche of the bone marrow. The chemokine CXCL12, which is the ligand for CXCR4, has been reported to play a major role in this process. We therefore examined whether the expression of these chemokines on lymphocytes is altered by exercise. Within the bone marrow we found no change in the percentage of B cells (Figure 8A; STD: 22.8 ± 2.0 vs. RUN: 24.2 ± 1.1%, *n* = 3 experiments, *p* = 0.99), CD4^+^ T cells (Figure 8A; STD: 52.0 ± 1.9 vs. RUN: 44.4 ± 0.5%, *n* = 3 experiments, *p* = 0.10) or Tregs (Figure 8A; STD: 43.0 ± 1.4 vs. RUN: 47.3 ± 3.1%, *n* = 3 experiments, *p* = 0.4) expressing CCR9 following exercise. Similarly, no change in the expression of CXCR4 was observed in B cells (Figure 8B; STD: 74.4 ± 1.9 vs. RUN: 75.5 ± 1.0%, *n* = 3 experiments, *p* = 0.7), CD4^+^ T cells (Figure 8B; STD: 34 ± 2.6 vs. RUN: 26.8 ± 1.7 %, *n* = 3 experiments, *p* = 0.2) or Tregs (Figure 8B; STD: 24.8 ± 2.0 vs. RUN: 24.5 ± 1.3%, *n* = 3 experiments, *p* = 0.99) in the bone marrow. In the spleen, physical activity had no effect on the percentage of B cells (Figure 8C; STD: 7.0 ± 0.2 vs. RUN: 7.2 ± 0.6%, *n* = 3 experiments, *p* = 0.99), CD4^+^ T cells (Figure 8C; STD: 6.9 ± 0.6 vs. RUN: 7.7 ± 0.5%, *n* = 3, *p* = 0.4) or Tregs (Figure 8C; STD:16.7 ± 0.5 vs. RUN: 17.8 ± 1.2%, *n* = 3 experiments, *p* = 0.80) expressing CCR9. Likewise, no change in CXCR4 expression was observed in any of the populations isolated from the spleen (Figure 8D; B cells: STD: 42.4 ± 4.2 vs. RUN: 46.6 ± 1.3%, *p* = 0.7, CD4^+^ T cells: STD: 11.3 ± 3.5 vs. RUN: 12.4 ± 1.1%, *p* = 0.7, Treg: STD: 21.1 ± 5.3 vs. RUN: 25.3 ± 0.7%, *p* = 0.7, *n* = 3 experiments).

## Discussion

In this study we show that T cells provide a neuro-immunological cross-talk that is involved in the control of baseline adult hippocampal neurogenesis. In contrast, we found that lymphocytes are not required for the activity-dependent regulation of neurogenesis above that baseline. Such distinct modes of precursor cell proliferation (and induction of neurogenesis) had been suggested by previous studies which showed that the increase in proliferation induced by exercise is absent after blockade of insulin-like growth factor-1 (34), VEGF (11), serotonin (35) or the endocannabinoid system (12). The current study highlights the importance of this distinction between baseline control and activity-dependent regulation of neurogenesis, which in the latter case is not dependent on lymphocytes.

Although immune regulation of the brain is most widely studied in the context of injury or disease, there is increasing evidence for the involvement of peripheral immune cells in maintaining normal brain homeostasis. Despite the fact that, under physiological conditions, peripheral T cells are only found in very low numbers within the brain parenchyma, they appear to play an important role in maintaining baseline levels of adult neurogenesis. Ziv et al. first showed that adult neurogenesis was impaired in the hippocampus of immune-deficient mice and that this deficit could be rescued by T cells which recognized the brain-specific antigen myelin basic protein (2). Studies from our own lab subsequently revealed that the general depletion of non-specific CD4^+^ T cells reduced adult hippocampal neurogenesis and impaired learning in the Morris water maze test (3). Our observation of a role of CD4^+^ but not CD8^+^ T cells was later corroborated independently by a large study involving more than 700 outbred mice (5).

Physical exercise is a strong physiological stimulus of adult hippocampal neurogenesis (8, 9, 25). Given the nature of the stimulus, which has been associated with immune effects (36), we hypothesized that the activation of peripheral immune cells, including T cells, may at least in part underlie this response. We had previously reported that mice lacking T cells, either by anti-CD4 antibody treatment or by CD4 transgenic knock-out, showed a similar increase in hippocampal neurogenesis in response to physical activity to that of WT mice (3). However, the normal pro-neurogenic effect of physical activity was absent in mice lacking the common γ-chain on the RAG2^−/−^ background, and therefore devoid of functional T, B, and NK cells (3). This is in contrast to the current study in which we observed a significant increase in neural precursor proliferation in the hippocampus of Rag2^−/−^γc^−/−^ mice following exercise. Given that the mean increase following exercise in the two reports was similar (34.37 vs. 32.24%), it is possible that the earlier study was underpowered.

Further strengthening our conclusion that the above populations of lymphocytes are not required for the physical activity-mediated increase in hippocampal precursor proliferation, we found that the distributions of CD4^+^ and CD8^+^ T cells, and Tregs, as well as B cells, were not altered in the bone marrow or peripheral lymphoid organs after exercise. In addition, we did not detect changes in the activation or maturation state of T cells in the peripheral lymphoid organs, and nor did we observe differential expression of chemokine receptors in any of the organs examined. Interestingly, we found an increased proportion of naive CD8^+^ T cells and a decrease in the effector memory compartment of CD8^+^ T cells in the bone marrow, indicating a probable impact of exercise on the maturation of bone marrow CD8^+^ T cells. Although we did not detect a change in the proportion of the different T cell sub-types following exercise, we cannot rule out a net increase in T cell number, as has been previously described (37). We also cannot exclude the potential impact of the chemokine receptors expressed in the brain on the recruitment of T cells, as Tregs have been shown to migrate to the injury site following stroke (38–40). In a model of multiple sclerosis such infiltrating myelin-specific T cells were able to stimulate oligodendrogenesis from resident parenchymal precursor cells, further indicating that such interactions are possible (41).

Although few T cells are found within the brain parenchyma, we investigated whether soluble factors secreted by large populations of peripheral T cells, either in the resting state or following stimulation, are capable of affecting neural stem cell proliferation. We found no effect of any of the T cell populations tested on adult hippocampal precursor cell proliferation. However, this does not exclude the possibility that T cells affect neural precursor proliferation *in vivo* where the signaling molecules secreted by these cells can interact with other signals present in the neural stem cell niche. In contrast, Wang et al. have shown that stimulated, but not unstimulated Tregs increase embryonic cortical cell proliferation *in vitro* via interleukin-10 signaling (24). They also reported enhanced *in vivo* proliferation of precursor cells in the SVZ after stroke, although their quantification was limited to a very small subregion. The observation by Wang et al. is consistent with numerous studies in that the activity of CD4^+^Foxp3^+^ Tregs is strictly dependent on their activation status, both *in vitro* and *in vivo*. This is probably best exemplified by standard co-culture assays, in which steady state Tregs (i.e., in the absence of TCR-mediated Treg stimulation) fail to suppress the activity of TCR-stimulated conventional CD4^+^ T cells. While the strict dependence of Treg activity on T cell receptor (re-)stimulation is key to prevent indiscriminate immunosuppression *in vivo*, the underlying molecular mechanisms have remained unclear. However, it is clear that the mere expression of activation markers (such as CD25) does not allow any conclusions on function. In fact, Tregs are characterized by the constitutive expression of a set of activation markers whose expression is only transiently induced in conventional CD4^+^ T cells (such as CD25), upon productive T cell receptor-mediated activation. The constitutive expression of such activation markers has been attributed to the molecular action of Foxp3, which directly binds to the respective promoter regions, thereby stabilizing and amplifying the expression of gene targets (42).

In summary, our findings further support the existence of a neuro-immunological cross-talk in adult hippocampal neurogenesis, and hence physiological brain plasticity. The data also support the idea that the homeostatic baseline control of adult neurogenesis differs from exercise-induced regulation, which does not involve T cells. This does not exclude the existence of neuro-immunological mechanisms involved in the activity-dependent regulation of adult neurogenesis. Importantly, however, our results further support the idea that multiple signaling systems are involved in controlling and regulating precursor cell proliferation and adult hippocampal neurogenesis.

## Ethics Statement

This study was carried out in accordance with the recommendations of the local ethics committee (Landesdirektion Sachsen) and in accordance with the European and national regulations (Tierschutzgesetz).

## Author Contributions

TW, SS, and GK: conceptualization and writing the original draft. TW and SS: formal analysis. TW, SS, NR, RR, and LG: investigation, TW, SS, GK, and KK: reviewing and editing the manuscript, and funding acquisition. TW, GK, and KK: supervision.

### Conflict of Interest Statement

The authors declare that the research was conducted in the absence of any commercial or financial relationships that could be construed as a potential conflict of interest.

## Supplementary Material

Supplementary Figure 1 The activation status of CD4+ T cells **(A)**, native T cells **(B)** and Tregs **(C)** was confirmed using flow cytometry.

Supplementary Raw Data This file contains all of the raw data that was analyzed and presented in this manuscript.
