# Supplementary material for: T Lymphocytes Contribute to the Control of Baseline Neural Precursor Cell Proliferation but Not the Exercise-Induced Up-Regulation of Adult Hippocampal Neurogenesis
Source: Front Immunol. 2018 Dec 11;9:2856. doi: 10.3389/fimmu.2018.02856 (PMC6297802; doi:10.3389/fimmu.2018.02856)
Supplement: Supplementary Figure 1 — The activation status of CD4+ T cells (A), native T cells (B) and Tregs (C) was confirmed using flow cytometry. [file Data_Sheet_1.PDF]

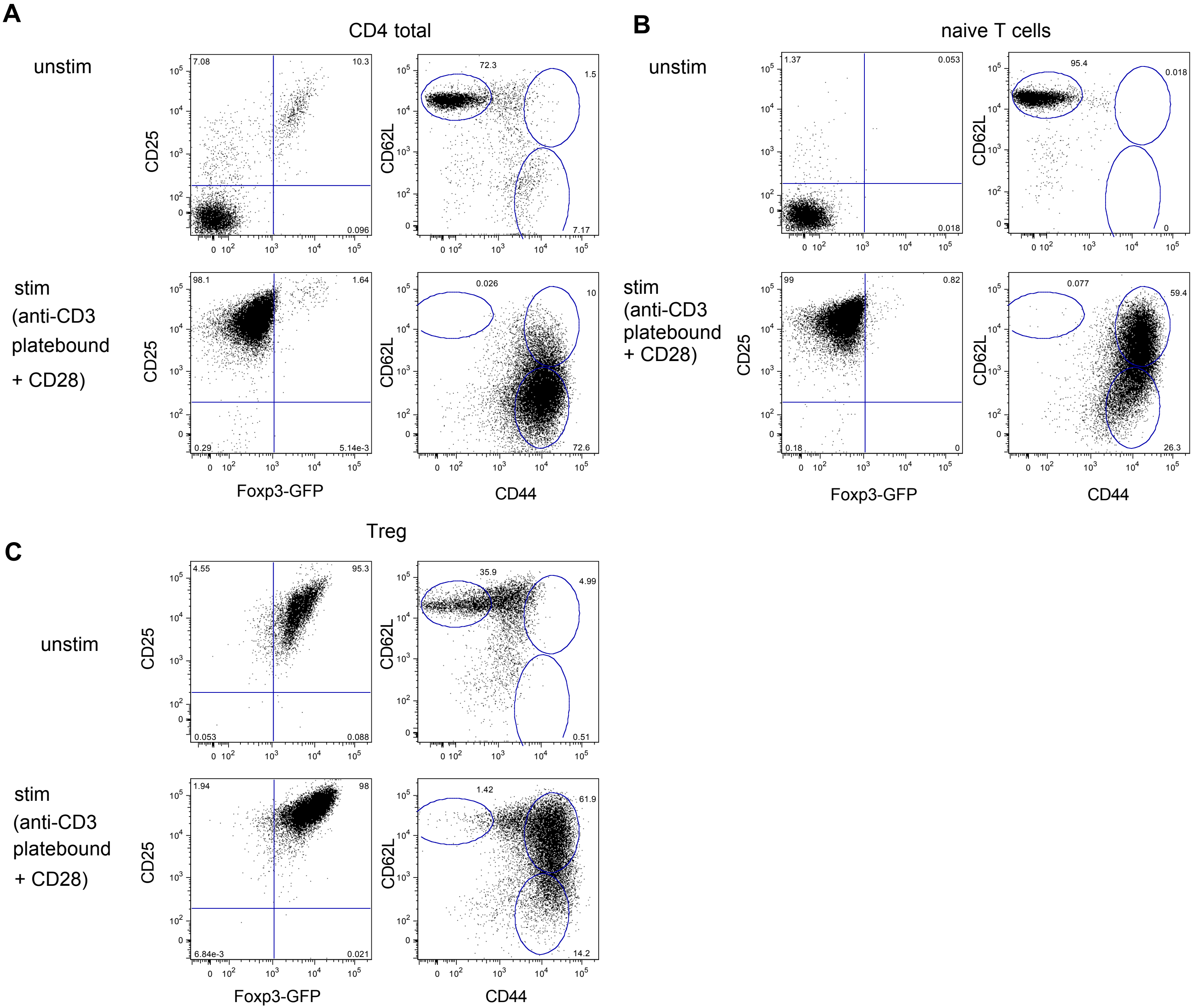
A
CD4 total
unstim
stim
(anti-CD3
platebound
+ CD28)
CD25
CD62L
Foxp3-GFP
CD44
7.08
10.3
0.096
72.3
1.5
7.17
98.1
1.64
0.29
5.14e-3
0.026
10
72.6
B
naive T cells
unstim
stim
(anti-CD3
platebound
+ CD28)
CD25
CD62L
Foxp3-GFP
CD44
1.37
0.053
0.018
95.4
0.018
0
99
0.82
0.18
0
0.077
59.4
26.3
C
Treg
unstim
stim
(anti-CD3
platebound
+ CD28)
CD25
CD62L
Foxp3-GFP
CD44
4.55
95.3
0.053
0.088
35.9
4.99
0.51
1.94
98
6.84e-3
0.021
1.42
61.9
14.2
