# Supplementary material for: T Lymphocytes Contribute to the Control of Baseline Neural Precursor Cell Proliferation but Not the Exercise-Induced Up-Regulation of Adult Hippocampal Neurogenesis
Source: Front Immunol. 2018 Dec 11;9:2856. doi: 10.3389/fimmu.2018.02856 (PMC6297802; doi:10.3389/fimmu.2018.02856)
Supplement: Supplementary Raw Data — This file contains all of the raw data that was analyzed and presented in this manuscript. [file Table_1.DOCX]

Supplementary data _ All raw data

Raw data Figure 1C Ki67+ cells per SGZ

| WT + DT | Saline | DT |
| --- | --- | --- |
| \| 5652 \| \| --- \| \| 2340 \| \| 2136 \| \| 3552 \| \| 4452 \| | \| 3624 \| \| --- \| \| 3564 \| \| 1980 \| \| 4260 \| \| 3390 \| \| 2226 \| \| 5106 \| \| 4380 \| \| 4908 \| \| 4224 \| \| 3540 \| \| 4188 \| | \| 6216 \| \| --- \| \| 4224 \| \| 2688 \| \| 4032 \| \| 2016 \| \| 3606 \| \| 3780 \| \| 3510 \| \| 6276 \| \| 6768 \| \| 7740 \| \| 7176 \| \| 6540 \| \| 4032 \| \| 4824 \| \| 5940 \| |

Raw data Figure 2B-F Proliferating cells per DG

| C57 STD | C57 RUN | Dereg STD | Dereg RUN | TCRa  STD | TCRa RUN | Rag1 STD | Rag1 RUN | Rag2 STD | Rag2 RUN |
| --- | --- | --- | --- | --- | --- | --- | --- | --- | --- |
| \| 3576 \| \| --- \| \| 3276 \| \| 3036 \| \| 2856 \| \| 3732 \| \| 3504 \| \| 4512 \| \| 4272 \| \| 4116 \| \| 4152 \| | \| 4632 \| \| --- \| \| 2976 \| \| 4584 \| \| 3696 \| \| 4164 \| \| 5280 \| \| 4452 \| \| 4716 \| \| 4356 \| \| 5388 \| | \| 2106 \| \| --- \| \| 2604 \| \| 1692 \| \| 3216 \| \| 3180 \| \| 3216 \| \| 3078 \| \| 1596 \| | \| 6936 \| \| --- \| \| 5910 \| \| 3720 \| \| 2946 \| \| 3378 \| \| 3162 \| \| 2796 \| \| 3648 \| | \| 3888 \| \| --- \| \| 3564 \| \| 3600 \| \| 4416 \| \| 4596 \| \| 4248 \| \| 5160 \| \| 4464 \| \| 3984 \| | \| 3708 \| \| --- \| \| 5268 \| \| 6108 \| \| 4932 \| \| 5316 \| \| 4740 \| \| 5220 \| | \| 4248 \| \| --- \| \| 2988 \| \| 3792 \| \| 3120 \| \| 3384 \| \| 3024 \| \| 3288 \| \| 3660 \| \| 2400 \| \| 3636 \| | \| 3816 \| \| --- \| \| 2928 \| \| 4548 \| \| 3384 \| \| 4224 \| \| 2868 \| \| 4236 \| \| 3648 \| \| 4788 \| \| 5016 \| \| 4200 \| | \| 2880 \| \| --- \| \| 1932 \| \| 3120 \| \| 3576 \| \| 3012 \| \| 2160 \| \| 2592 \| \| 3012 \| \| 3840 \| | \| 4008 \| \| --- \| \| 4176 \| \| 3672 \| \| 2448 \| \| 3864 \| \| 3252 \| \| 6048 \| \| 3240 \| |

Raw data Figure 2H-K Proliferating cells per SVZ

| C57 STD | C57 RUN | TCRa  STD | TCRa RUN | Rag1 STD | Rag1 RUN | Rag2 STD | Rag2 RUN |
| --- | --- | --- | --- | --- | --- | --- | --- |
| \| 21468 \| \| --- \| \| 20076 \| \| 20556 \| \| 24672 \| \| 12504 \| \| 18912 \| \| 6780 \| \| 9612 \| | \| 22944 \| \| --- \| \| 17940 \| \| 17640 \| \| 21732 \| \| 23256 \| \| 17388 \| \| 11748 \| \| 22020 \| \| 16812 \| \| 14652 \| | \| 21516 \| \| --- \| \| 17712 \| \| 15144 \| \| 21648 \| \| 17292 \| \| 18924 \| \| 21768 \| \| 19248 \| \| 21468 \| | \| 18444 \| \| --- \| \| 19716 \| \| 21648 \| \| 20412 \| \| 20028 \| \| 18120 \| \| 25956 \| \| 24564 \| \| 22296 \| | \| 20424 \| \| --- \| \| 20376 \| \| 18804 \| \| 21036 \| \| 16248 \| \| 23088 \| \| 20856 \| \| 5808 \| \| 14580 \| | \| 18612 \| \| --- \| \| 15684 \| \| 19440 \| \| 18888 \| \| 18288 \| \| 21792 \| \| 16560 \| \| 19068 \| \| 19320 \| \| 16368 \| | \| 24084 \| \| --- \| \| 21732 \| \| 13884 \| \| 16632 \| \| 20184 \| \| 23064 \| \| 13092 \| \| 18360 \| | \| 20064 \| \| --- \| \| 15336 \| \| 20172 \| \| 21996 \| \| 22080 \| \| 23808 \| \| 24240 \| \| 19668 \| \| 16404 \| |

Raw data Figure 3B Neurosphere number

| NSC only | CD4+ s | Naïve Ts | Treg s | CD4+ us | Naïve T us | Treg us |
| --- | --- | --- | --- | --- | --- | --- |
| \| 9 \| \| --- \| \| 7 \| \| 14 \| \| 15 \| \| 38 \| \| 6 \| \| 11 \| \| 13 \| \| 16 \| \| 19 \| \| 9 \| \| 6 \| \| 3 \| \| 6 \| | \| 51 \| \| --- \| \| 9 \| \| 11 \| \| 7 \| \| 6 \| \| 5 \| | \| 15 \| \| --- \| \| 16 \| \| 8 \| \| 0 \| \| 4 \| \| 5 \| | \| 10 \| \| --- \| \| 6 \| \| 5 \| \| 5 \| \| 5 \| | \| 9 \| \| --- \| \| 19 \| \| 10 \| \| 14 \| \| 8 \| \| 19 \| \| 10 \| \| 6 \| \| 9 \| \| 8 \| | \| 11 \| \| --- \| \| 12 \| \| 17 \| \| 22 \| \| 4 \| \| 9 \| \| 3 \| \| 3 \| \| 3 \| \| 6 \| | \| 20 \| \| --- \| \| 10 \| \| 12 \| \| 9 \| \| 7 \| \| 7 \| \| 3 \| \| 7 \| \| 6 \| |

Raw data Figure 3C Neurosphere size

| NSC only | CD4+ stim | Naïve T stim | Treg stim | CD4+ unstim | Naïve T unstim | Treg unstim |
| --- | --- | --- | --- | --- | --- | --- |
| \| 100 \| \| --- \| \| 70 \| \| 60 \| \| 50 \| \| 40 \| \| 40 \| \| 50 \| \| 70 \| \| 60 \| \| 70 \| \| 110 \| \| 50 \| \| 70 \| \| 50 \| \| 100 \| \| 90 \| \| 60 \| \| 70 \| \| 80 \| \| 70 \| \| 80 \| \| 70 \| \| 70 \| \| 70 \| \| 70 \| \| 50 \| \| 60 \| \| 40 \| \| 60 \| \| 60 \| \| 60 \| \| 60 \| \| 70 \| \| 70 \| \| 50 \| \| 70 \| \| 50 \| \| 40 \| \| 50 \| \| 40 \| \| 50 \| \| 50 \| \| 40 \| \| 80 \| \| 140 \| \| 90 \| \| 70 \| \| 40 \| \| 50 \| \| 40 \| \| 40 \| \| 60 \| \| 60 \| \| 70 \| \| 70 \| \| 50 \| \| 70 \| \| 40 \| \| 50 \| \|  \| | \| 50 \| \| --- \| \| 100 \| \| 70 \| \| 60 \| \| 80 \| \| 50 \| \| 70 \| \| 100 \| \| 50 \| \| 50 \| \| 50 \| \| 70 \| \| 60 \| \| 90 \| \| 50 \| \| 50 \| \| 50 \| \| 60 \| \| 60 \| \| 70 \| \| 80 \| \| 50 \| \| 70 \| \| 50 \| \| 90 \| \| 60 \| \| 40 \| \| 50 \| \| 50 \| | \| 60 \| \| --- \| \| 80 \| \| 80 \| \| 50 \| \| 90 \| \| 50 \| \| 100 \| \| 80 \| \| 50 \| \| 50 \| \| 60 \| \| 80 \| \| 40 \| \| 40 \| \| 70 \| \| 70 \| \| 80 \| | \| 50 \| \| --- \| \| 60 \| \| 90 \| \| 60 \| \| 90 \| \| 60 \| \| 80 \| \| 50 \| \| 50 \| \| 40 \| \| 40 \| \| 100 \| \| 60 \| \| 60 \| \| 60 \| \| 50 \| | \| 50 \| \| --- \| \| 80 \| \| 50 \| \| 40 \| \| 100 \| \| 50 \| \| 50 \| \| 40 \| \| 40 \| \| 70 \| \| 60 \| \| 50 \| \| 50 \| \| 70 \| \| 40 \| \| 40 \| \| 80 \| \| 90 \| \| 100 \| \| 80 \| \| 100 \| \| 50 \| \| 40 \| \| 50 \| \| 50 \| | \| 50 \| \| --- \| \| 40 \| \| 60 \| \| 100 \| \| 50 \| \| 70 \| \| 60 \| \| 40 \| \| 60 \| \| 170 \| \| 90 \| \| 100 \| \| 70 \| \| 40 \| \| 40 \| | \| 80 \| \| --- \| \| 40 \| \| 40 \| \| 40 \| \| 90 \| \| 90 \| \| 40 \| \| 80 \| \| 70 \| \| 40 \| \| 50 \| \| 40 \| \| 40 \| \| 90 \| \| 80 \| \| 70 \| \| 60 \| |

Raw data Figure 4A B cells

| BM  STD | BM RUN | scLN STD | scLN RUN | mLN STD | mLN  RUN | Spleen STD | Spleen RUN |
| --- | --- | --- | --- | --- | --- | --- | --- |
| \| 29.7 \| \| --- \| \| 27.3 \| \| 25.8 \| \| 25.6 \| \| 31.6 \| \| 25.3 \| | \| 24.3 \| \| --- \| \| 21.6 \| \| 22.6 \| \| 26.3 \| \| 23.3 \| \| 30.3 \| | \| 17.6 \| \| --- \| \| 24.8 \| \| 18.7 \| \| 15 \| \| 12.9 \| \| 13.3 \| | \| 19.9 \| \| --- \| \| 20.4 \| \| 21.8 \| \| 10.4 \| \| 10.7 \| \| 15.7 \| | \| 18.5 \| \| --- \| \| 18.3 \| \| 18.2 \| \| 15.8 \| \| 11.9 \| \| 15.4 \| | \| 19.5 \| \| --- \| \| 17 \| \| 17.3 \| \| 16.6 \| \| 13.2 \| \| 18.3 \| | \| 57.2 \| \| --- \| \| 60.1 \| \| 55.9 \| \| 39.9 \| \| 40.4 \| \| 39 \| | \| 58.2 \| \| --- \| \| 55.7 \| \| 56.5 \| \| 40.6 \| \| 38.2 \| \| 43 \| |

Raw data Figure 4B CD8+ T cells

| BM  STD | BM RUN | scLN STD | scLN RUN | mLN STD | mLN  RUN | Spleen STD | Spleen RUN |
| --- | --- | --- | --- | --- | --- | --- | --- |
| \| 3.85 \| \| --- \| \| 3.95 \| \| 4.16 \| \| 2.7 \| \| 2.5 \| \| 2.8 \| | \| 4.13 \| \| --- \| \| 3.6 \| \| 2.76 \| \| 3.7 \| \| 4.5 \| \| 3.3 \| | \| 27.7 \| \| --- \| \| 28.3 \| \| 25.9 \| \| 22 \| \| 23.5 \| \| 22.1 \| | \| 30.5 \| \| --- \| \| 31.5 \| \| 31.1 \| \| 22.4 \| \| 26.6 \| \| 18.6 \| | \| 31 \| \| --- \| \| 27.9 \| \| 31.7 \| \| 24.2 \| \| 26.4 \| \| 30.6 \| | \| 33.2 \| \| --- \| \| 33.9 \| \| 32.6 \| \| 30.9 \| \| 31.9 \| \| 23.4 \| | \| 12.4 \| \| --- \| \| 11.5 \| \| 14.5 \| \| 10.1 \| \| 10.5 \| \| 11.6 \| | \| 12.8 \| \| --- \| \| 15.7 \| \| 13.9 \| \| 10.9 \| \| 12.1 \| \| 8.7 \| |

Raw data Figure 4C CD4+ T cells

| BM  STD | BM RUN | scLN STD | scLN RUN | mLN STD | mLN  RUN | Spleen STD | Spleen RUN |
| --- | --- | --- | --- | --- | --- | --- | --- |
| \| 2.2 \| \| --- \| \| 2.27 \| \| 2.21 \| \| 1.3 \| \| 1.6 \| \| 2.1 \| | \| 2.07 \| \| --- \| \| 2.45 \| \| 1.86 \| \| 2.9 \| \| 3.4 \| \| 2.8 \| | \| 47.4 \| \| --- \| \| 43.5 \| \| 45.6 \| \| 47.2 \| \| 45 \| \| 42 \| | \| 42.2 \| \| --- \| \| 41.6 \| \| 41.3 \| \| 44.4 \| \| 43.8 \| \| 43.8 \| | \| 50.3 \| \| --- \| \| 49.4 \| \| 52.3 \| \| 50.9 \| \| 49.7 \| \| 47.6 \| | \| 46 \| \| --- \| \| 47.3 \| \| 47.2 \| \| 47.4 \| \| 46 \| \| 48.6 \| | \| 24.1 \| \| --- \| \| 22.5 \| \| 22.8 \| \| 25.5 \| \| 22.1 \| \| 22.3 \| | \| 21.5 \| \| --- \| \| 20.7 \| \| 22.6 \| \| 23.3 \| \| 21.3 \| \| 21.8 \| |

Raw data Figure 4D Tregs

| BM  STD | BM RUN | scLN STD | scLN RUN | mLN STD | mLN  RUN | Spleen STD | Spleen RUN |
| --- | --- | --- | --- | --- | --- | --- | --- |
| \| 23.3 \| \| --- \| \| 22.7 \| \| 22.4 \| \| 27.8 \| \| 23.4 \| \| 21.4 \| | \| 24.8 \| \| --- \| \| 20.4 \| \| 23.1 \| \| 19.7 \| \| 17.6 \| \| 23.6 \| | \| 6.94 \| \| --- \| \| 8.52 \| \| 7.43 \| \| 5.8 \| \| 7.2 \| \| 7.2 \| | \| 8.32 \| \| --- \| \| 7.42 \| \| 7.57 \| \| 6.5 \| \| 7.6 \| \| 6.7 \| | \| 7.74 \| \| --- \| \| 8.12 \| \| 6.95 \| \| 6 \| \| 6.2 \| \| 6.8 \| | \| 8.4 \| \| --- \| \| 8.37 \| \| 8.71 \| \| 6.1 \| \| 7 \| \| 6.3 \| | \| 8.19 \| \| --- \| \| 7.24 \| \| 7.45 \| \| 5.6 \| \| 5.7 \| \| 6.4 \| | \| 9.72 \| \| --- \| \| 8.54 \| \| 9.05 \| \| 5.5 \| \| 6.2 \| \| 6.6 \| |

Raw data Figure 5B Spleen CD8+ T cells

| Naïve STD | Naïve RUN | TCM STD | TCM RUN | TEM STD | TEM RUN |
| --- | --- | --- | --- | --- | --- |
| \| 81.3 \| \| --- \| \| 80.2 \| \| 81.6 \| \| 72 \| \| 66.4 \| \| 68.1 \| | \| 75.8 \| \| --- \| \| 80.3 \| \| 80.3 \| \| 67.9 \| \| 59.4 \| \| 68.5 \| | \| 5.15 \| \| --- \| \| 6.02 \| \| 7.2 \| \| 12.3 \| \| 9.5 \| \| 10.9 \| | \| 7.71 \| \| --- \| \| 8.07 \| \| 7.76 \| \| 10.1 \| \| 8.4 \| \| 10.3 \| | \| 1.12 \| \| --- \| \| 1.61 \| \| 0.9 \| \| 1.4 \| \| 1.9 \| \| 1.3 \| | \| 1.55 \| \| --- \| \| 0.71 \| \| 0.86 \| \| 2.1 \| \| 1.7 \| \| 1.5 \| |

Raw data Figure 5D Spleen CD4+ T cells

| Naïve STD | Naïve RUN | Memory STD | Memory RUN |
| --- | --- | --- | --- |
| \| 83.8 \| \| --- \| \| 82.1 \| \| 85.2 \| \| 87.1 \| \| 83.5 \| \| 78.5 \| | \| 78.6 \| \| --- \| \| 79.7 \| \| 79.2 \| \| 79.5 \| \| 69.2 \| \| 80.4 \| | \| 8.52 \| \| --- \| \| 9.07 \| \| 6.81 \| \| 4.9 \| \| 6.5 \| \| 10.9 \| | \| 10.3 \| \| --- \| \| 10.5 \| \| 11.3 \| \| 8.6 \| \| 8.1 \| \| 7.8 \| |

Raw data Figure 5G,H Spleen Treg

| Treg STD | Treg RUN | Naïve Treg STD | Naïve Treg RUN | Memory Treg STD | Memory Treg RUN |
| --- | --- | --- | --- | --- | --- |
| \| 8.19 \| \| --- \| \| 7.24 \| \| 7.45 \| \| 5.6 \| \| 5.7 \| \| 6.4 \| | \| 9.72 \| \| --- \| \| 8.54 \| \| 9.05 \| \| 5.5 \| \| 6.2 \| \| 6.6 \| | \| 55.9 \| \| --- \| \| 49 \| \| 53.6 \| \| 66.4 \| \| 65 \| \| 56 \| | \| 43.7 \| \| --- \| \| 47.4 \| \| 42.9 \| \| 56.4 \| \| 46.7 \| \| 56.4 \| | \| 17.8 \| \| --- \| \| 21.9 \| \| 17 \| \| 14.4 \| \| 14.9 \| \| 19.7 \| | \| 21.9 \| \| --- \| \| 20.1 \| \| 25.6 \| \| 22.2 \| \| 24.7 \| \| 21.5 \| |

Raw data Figure 6B scLN CD8+ T cells

| Naïve STD | Naïve RUN | TCM STD | TCM RUN | TEM STD | TEM RUN |
| --- | --- | --- | --- | --- | --- |
| \| 84.9 \| \| --- \| \| 82.8 \| \| 83.2 \| \| 76.7 \| \| 71.9 \| \| 73.5 \| | \| 82.9 \| \| --- \| \| 82.6 \| \| 83.6 \| \| 70.7 \| \| 71.3 \| \| 74.4 \| | \| 4.04 \| \| --- \| \| 5.14 \| \| 5.46 \| \| 8.2 \| \| 8.5 \| \| 8.5 \| | \| 5.05 \| \| --- \| \| 5.51 \| \| 5.91 \| \| 10.2 \| \| 8.6 \| \| 8.1 \| | \| 0.31 \| \| --- \| \| 0.44 \| \| 0.37 \| \| 0.3 \| \| 0.4 \| \| 0.4 \| | \| 0.33 \| \| --- \| \| 0.4 \| \| 0.36 \| \| 0.5 \| \| 0.4 \| \| 0.3 \| |

Raw data Figure 6D scLN CD4+ T cells

| Naïve STD | Naïve RUN | Memory STD | Memory RUN |
| --- | --- | --- | --- |
| \| 88.9 \| \| --- \| \| 89.1 \| \| 88 \| \| 90.7 \| \| 87.3 \| \| 88.1 \| | \| 88.6 \| \| --- \| \| 88 \| \| 87.9 \| \| 88.6 \| \| 87.9 \| \| 87.2 \| | \| 2.08 \| \| --- \| \| 1.86 \| \| 2.09 \| \| 1 \| \| 1.4 \| \| 1.5 \| | \| 2.11 \| \| --- \| \| 2.21 \| \| 2.56 \| \| 1.3 \| \| 1.6 \| \| 1.6 \| |

Raw data Figure 6G,H Tregs

| Treg STD | Treg RUN | Naïve Treg STD | Naïve Treg RUN | Memory Treg STD | Memory Treg RUN |
| --- | --- | --- | --- | --- | --- |
| \| 6.94 \| \| --- \| \| 8.52 \| \| 7.43 \| \| 5.8 \| \| 7.2 \| \| 7.2 \| | \| 8.32 \| \| --- \| \| 7.42 \| \| 7.57 \| \| 6.5 \| \| 7.6 \| \| 6.7 \| | \| 59 \| \| --- \| \| 65.9 \| \| 62 \| \| 67.2 \| \| 60.6 \| \| 63.1 \| | \| 61.3 \| \| --- \| \| 55.1 \| \| 53.9 \| \| 63.8 \| \| 67.4 \| \| 63.3 \| | \| 15.2 \| \| --- \| \| 10.2 \| \| 13.3 \| \| 10.3 \| \| 12.6 \| \| 12 \| | \| 13.2 \| \| --- \| \| 16.4 \| \| 17.9 \| \| 11.3 \| \| 10.6 \| \| 10.5 \| |

Raw data Figure 7B Bone marrow CD8+ T cells

| Naïve STD | Naïve RUN | TCM STD | TCM RUN | TEM STD | TEM RUN |
| --- | --- | --- | --- | --- | --- |
| \| 50.5 \| \| --- \| \| 48.2 \| \| 47.2 \| \| 55.9 \| \| 54.6 \| \| 55.7 \| | \| 59.5 \| \| --- \| \| 55.9 \| \| 52.3 \| \| 59.6 \| \| 61.6 \| \| 59 \| | \| 9.45 \| \| --- \| \| 8.49 \| \| 6.24 \| \| 19 \| \| 18.5 \| \| 18.5 \| | \| 19.3 \| \| --- \| \| 18.9 \| \| 17.3 \| \| 15.7 \| \| 16.3 \| \| 17.5 \| | \| 6.3 \| \| --- \| \| 4.48 \| \| 5.95 \| \| 4.7 \| \| 4.3 \| \| 3.3 \| | \| 2.6 \| \| --- \| \| 3.54 \| \| 3.45 \| \| 3.2 \| \| 2.8 \| \| 2.7 \| |

Raw Data Figure 7D Bone marrow CD4+ T cells

| Naïve STD | Naïve RUN | Memory STD | Memory RUN |
| --- | --- | --- | --- |
| \| 57 \| \| --- \| \| 46.4 \| \| 66.8 \| \| 41.6 \| \| 44 \| \| 41.4 \| | \| 45.4 \| \| --- \| \| 38.7 \| \| 47.6 \| \| 67.1 \| \| 67.2 \| \| 55.9 \| | \| 26.2 \| \| --- \| \| 37.1 \| \| 18.5 \| \| 33.8 \| \| 30.5 \| \| 36.5 \| | \| 38.4 \| \| --- \| \| 45.9 \| \| 34.7 \| \| 18.8 \| \| 18.6 \| \| 22.2 \| |

Raw data Figure 7G,H Bone marrow Tregs

| Treg STD | Treg RUN | Naïve Treg STD | Naïve Treg RUN | Memory Treg STD | Memory Treg RUN |
| --- | --- | --- | --- | --- | --- |
| \| 23.3 \| \| --- \| \| 22.7 \| \| 22.4 \| \| 27.8 \| \| 23.4 \| \| 21.4 \| | \| 24.8 \| \| --- \| \| 20.4 \| \| 23.1 \| \| 19.7 \| \| 17.6 \| \| 23.6 \| | \| 54.5 \| \| --- \| \| 54.5 \| \| 67.6 \| \| 50.9 \| \| 58.1 \| \| 62.6 \| | \| 46.6 \| \| --- \| \| 43.7 \| \| 37 \| \| 75.6 \| \| 76.7 \| \| 59.1 \| | \| 17.4 \| \| --- \| \| 19.6 \| \| 7.86 \| \| 18.2 \| \| 10.1 \| \| 12.6 \| | \| 24.5 \| \| --- \| \| 26.6 \| \| 30.3 \| \| 7.2 \| \| 7.1 \| \| 15.1 \| |

Raw data Figure 8A Percentage CCR9 cells in bone marrow

| B cell STD | B cell RUN | T cell STD | T cell RUN | Treg STD | Treg RUN |
| --- | --- | --- | --- | --- | --- |
| \| 23.6 \| \| --- \| \| 25.7 \| \| 19 \| | \| 26.3 \| \| --- \| \| 23.2 \| \| 23 \| | \| 54.8 \| \| --- \| \| 52.7 \| \| 48.4 \| | \| 45.1 \| \| --- \| \| 44.8 \| \| 43.4 \| | \| 44.1 \| \| --- \| \| 44.6 \| \| 40.2 \| | \| 50.7 \| \| --- \| \| 50 \| \| 41.1 \| |

Raw data Figure 8B Percentage CXCR4 cells in bone marrow

| B cell STD | B cell RUN | T cell STD | T cell RUN | Treg STD | Treg RUN |
| --- | --- | --- | --- | --- | --- |
| \| 71.9 \| \| --- \| \| 78.2 \| \| 73.2 \| | \| 75.6 \| \| --- \| \| 77.2 \| \| 73.6 \| | \| 38.9 \| \| --- \| \| 33.2 \| \| 29.9 \| | \| 24.9 \| \| --- \| \| 25.3 \| \| 30.1 \| | \| 28.2 \| \| --- \| \| 25 \| \| 21.3 \| | \| 24.1 \| \| --- \| \| 27 \| \| 22.5 \| |

Raw data Figure 8C Percentage CCR9 cells in spleen

| B cell STD | B cell RUN | T cell STD | T cell RUN | Treg STD | Treg RUN |
| --- | --- | --- | --- | --- | --- |
| \| 6.7 \| \| --- \| \| 7.5 \| \| 6.9 \| | \| 8.1 \| \| --- \| \| 6.2 \| \| 7.3 \| | \| 6 \| \| --- \| \| 6.5 \| \| 8.1 \| | \| 7.9 \| \| --- \| \| 8.5 \| \| 6.7 \| | \| 17.6 \| \| --- \| \| 15.8 \| \| 16.8 \| | \| 17.5 \| \| --- \| \| 20 \| \| 15.8 \| |

Raw data Figure 8D Percentage CXCR4 cells in spleen

| B cell STD | B cell RUN | T cell STD | T cell RUN | Treg STD | Treg RUN |
| --- | --- | --- | --- | --- | --- |
| \| 37.2 \| \| --- \| \| 39.2 \| \| 50.8 \| | \| 44.5 \| \| --- \| \| 49 \| \| 46.4 \| | \| 7.5 \| \| --- \| \| 8.1 \| \| 18.4 \| | \| 10.4 \| \| --- \| \| 12.7 \| \| 14 \| | \| 14.7 \| \| --- \| \| 17 \| \| 31.6 \| | \| 24 \| \| --- \| \| 25.8 \| \| 26.2 \| |
